# Factors influencing women’s decision-making regarding complementary medicine product use in pregnancy and lactation

**DOI:** 10.1186/s12884-019-2396-2

**Published:** 2019-08-07

**Authors:** Larisa Ariadne Justine Barnes, Lesley Barclay, Kirsten McCaffery, Parisa Aslani

**Affiliations:** 1 The University of Sydney, School of Pharmacy and University Centre for Rural Health, Faculty of Medicine and Health, PO Box 3074, Lismore, NSW 2480 Australia; 2 The University of Sydney, University Centre for Rural Health and Sydney School of Public Health, Faculty of Medicine and Health, Edward Ford Building (A27), Sydney, NSW 2006 Australia; 3 The University of Sydney, Sydney Health Literacy Lab, Sydney School of Public Health, Faculty of Medicine and Health, Edward Ford Building (A27), Sydney, NSW 2006 Australia; 4 The University of Sydney, School of Pharmacy, Faculty of Medicine and Health, Rm N502, Pharmacy & Bank Building (A15), Science Rd, Camperdown, NSW 2006 Australia

**Keywords:** Complementary therapies, Herbal medicine, Dietary supplements, Pregnancy, Breast feeding, Decision making, Information seeking behaviour, Health literacy, Qualitative research

## Abstract

**Background:**

The prevalence of complementary medicine product (CMP) use by pregnant or breastfeeding Australian mothers is high, however, there is limited data on factors influencing women’s decision-making to use CMPs. This study explored and described the factors influencing women’s decisions take a CMP when pregnant or breastfeeding.

**Methods:**

Qualitative in-depth interviews and focus group discussions were held with 25 pregnant and/or breastfeeding women who currently used CMPs. Participants’ health literacy was assessed using a validated single-item health literacy screening question and the Newest Vital Sign. Interview and focus group discussions were audio-recorded, transcribed verbatim and thematically analysed.

**Results:**

Participants were a homogenous group. Most had higher education, medium to high incomes and high health literacy skills. They actively sought information from multiple sources and used a reiterative collation and assessment process. Their decision-making to take or not to take CMPs was informed by the need to establish the safety of the CMPs, as well as possible benefits or harms to their baby’s or their own health that could result from taking a CMP. Their specific information needs included the desire to access comprehensive, consistent, clear, easy to understand, and evidence-based information. Women preferred to access information from reputable sources, namely, their trusted health care practitioners, and information linked to government or hospital websites and published research. A lack of comprehensive, clear, consistent, or evidence-based information often led to decisions not to take a CMP, as they felt unable to adequately establish its safety or benefits. Conversely, when the participants felt the CMPs information they collected was good quality and from reputable sources, it reassured them of the safety of the CMP in pregnancy and/or breastfeeding. If this confirmed a clear benefit to their baby or themselves, they were more likely to decide to take a CMP.

**Conclusions:**

The participants’ demographic profile confirms previous research concerning Australian women who use CMPs during pregnancy and lactation. Participants’ high health literacy skills led them to engage in a reiterative, information-seeking and analysis process fuelled by the need to find clear information before making the decision to take, or not to take, a CMP.

**Electronic supplementary material:**

The online version of this article (10.1186/s12884-019-2396-2) contains supplementary material, which is available to authorized users.

## Background

Australian women commonly use complementary medicine products (CMPs) such as herbal medicines (over 50% of women) and vitamin or mineral supplements (almost 90% of women) in pregnancy [1, 2]. Herbal medicine use in pregnancy ranges from 4 to 69% worldwide [2]. Australian breastfeeding women also commonly use herbal medicines [3] and herbal galactagogue use has been noted internationally also [4–6]. Some specific nutritional supplements like folic acid and iodine are recommended during pregnancy or breastfeeding [7] while women take other popular CMPs like multivitamins because they believe this will help them meet the additional nutritional requirements of pregnancy and lactation [8]. In general, consumers actively seek health information outside of health care consultations [9], and Australian mothers access a variety of information sources including additional health care professionals, other pregnant or breastfeeding mothers, and media, including the Internet [3, 10–12].

Self-determination in health care choices may influence pregnant or breastfeeding mothers’ choices to use CMPs [13–16], especially in relation to desiring holistic health care [15]. Common reasons given for mothers’ use of CMPs include **t**reatment of common conditions of pregnancy or lactation [3, 17, 18], to prepare for a natural childbirth [13, 16, 19], and to support and maintain health [20] or breastmilk production [3, 17, 21]. Other factors identified that have been associated with use of complementary therapies, including CMPs, in pregnancy include cultural factors, positive experiences with previous use, and perceptions that complementary medicines may be a safer choice than pharmaceutical medications [16, 22]. Many women feel a responsibility to bear healthy children, leading to balancing what they perceive as possible harms to the health of their unborn or breastfeeding babies and themselves while also considering their health care practitioners’ (HCPs’) advice [22]. This results in decisions to take or not to take CMPs and pharmaceutical medicines, primarily influenced by perceived possible harms to their babies [22].

Other research into decision-making around use of complementary and alternative medicine (CAM), including CMPs in pregnancy and lactation, has found that women’s decisions are influenced by personal beliefs and previous experiences of CAM due to difficulties in finding reliable information [16]. Perceptions of their HCPs’ personal beliefs regarding CAM, scope of practice, and time spent in consultation also influence women’s information-seeking behaviours [16]. While both biomedical and CAM HCPs may be consulted during pregnancy and lactation, women actively seek information on CMPs from their CAM HCPs whom they view as providing holistic care. Biomedical providers’ opinions on CMPs may be dismissed if they are perceived to have little expertise in CAM therapies and to either be against or uninformed about CAM therapies [16]. Time constraints of appointments with obstetricians and midwives are also frustrating for women wanting to discuss their pregnancies or breastfeeding difficulties, furthering their appreciation of time spent with CAM HCPs [16, 23]. Specific research into women’s choices to use CMPs in lactation, link decision-making to psychological benefits related to perceived and actual increases in breastmilk production, successful breastfeeding and self-care during the postpartum period [17, 21, 24–26].

Health literacy is defined as the ability to seek, find and understand health information, and use that information to make informed decisions [27–29]. Three main health literacy and information seeking related concerns are apparent in the literature when looking at women’s CMP use in pregnancy and lactation. These are 1) women’s difficulties in finding CMPs information [16]; 2) HCPs’ concerns with women’s reliance on lay information sources, and consequent self-prescription of CMPs [11, 16, 30]; and 3) concerns regarding women’s perceptions that CMPs are natural and therefore safe, when the safety profiles of many CMPs have not been established [11, 31]. A lack of confidence in analysing available information, and lack of evidence around CMP use in pregnancy and lactation has been expressed both in published research and by women themselves [16, 17, 32].

While the Australian prevalence of CMP use in pregnancy and lactation, and common reasons for its use have been investigated [1, 3, 12], women’s decision-making processes and factors that influence women to take or not to take CMPs during pregnancy and lactation have not been adequately explored [11, 13]. As part of a larger investigation into health literacy and decision-making processes pregnant and/or breastfeeding women use when choosing to use CMPs, this paper aims to explore and describe the factors that influence women’s decisions to take or not to take a CMP when pregnant or breastfeeding.

## Methods

The methods used in this research have been previously published in a related paper that reports different results [33]. Further information can also be found in Additional file 1.

### Operational definition of complementary medicine products (CMPs)

For the purposes of this research, CMPs were defined as herbal medicines in tea, capsule, tablet or ethanolic extract form [34, 35] ingested, or applied topically as creams or inhalations; vitamin and/or mineral micronutrient supplements; and food supplements (e.g. probiotics or protein powders) [36]. CMPs could be self-prescribed and purchased over-the-counter, or after consultation with a health care practitioner [37].

### Study design

A qualitative research design using semi-structured in-depth interviews (IDIs) and focus group discussions (FGDs) was used. The semi-structured nature of the questions guided the inquiry, but were flexible so participants could elaborate on information important to them, and allowed new information to arise [38]. Women’s perceptions, beliefs, values and motivations for CMP use when pregnant or breastfeeding were central to the inquiry, and qualitative methods enabled an appropriate investigation of these [38, 39]. The use of both interviews and focus group discussions allowed women to choose the format with which they were most comfortable, and the use of telephone or Skype interviews allowed women at a distance from the researcher to participate.

### Participants and recruitment

To be eligible for the study, women needed to be over the age of 18, currently pregnant and/or breastfeeding, and living in the Northern Rivers region or Sydney, New South Wales, or metropolitan areas of Brisbane or the Gold Coast in Queensland. These areas were chosen for proximity to the researchers. Participants also needed to be currently taking or have taken at least one CMP in the last year and have sufficient English-language skills to participate. Face-to-face IDIs and FGDs took place in public places comfortable for the mothers including public libraries, playgroup venues and community centres. All participants were given a $20 supermarket voucher to thank them for their participation.

Participants were initially recruited through purposive sampling, followed by snowball sampling. The lead author obtained permission to visit a range of groups such as antenatal classes, playgroups, pregnancy and postnatal yoga classes, and support groups in the Northern Rivers Region to briefly explain the study and leave information flyers. Posters and flyers were also displayed in local pharmacies and allied health practices. Additionally, the study was advertised on free local classified advertising networks for all included regions, and through [The Institution’s] electronic media channels and posters on campus. After 22 participants, and using concurrent analysis alongside data collection, thematic saturation was reached [40]. An additional three interviews were held to confirm that no new themes were evident.

### Data collection

The guiding theme for this investigation centred on women’s decision-making regarding CMP use in pregnancy and lactation. The open questions used to explore this theme are detailed in Table 1. These questions were refined after pre-testing for face and content validity with five women through a semi-structured interview (one pregnant woman), and a focus group discussion (one pregnant and three breastfeeding women). All IDIs and FGDs were audio recorded and transcribed by an independent transcription service. The lead author also kept a detailed research journal throughout the process to document ideas and themes as they became apparent.

**Table 1:** Questions used to guide semi-structured interviews and focus group discussions [33]^a^

1. Why do you use complementary medicine products?	
2. What sort of information do you want when considering taking complementary medicine products?	
3. What sort of information do you feel women who are pregnant or lactating need when considering using complementary medicine products?	
4. Where do you find the information you need when choosing to use complementary medicine products in pregnancy or whilst breastfeeding? What resources do you use?	
5. What do you feel would help pregnant and lactating women get the complementary medicines information they want and need to make safe decisions regarding using complementary medicine products?	
6. How easy is it for you to understand the information about complementary medicines you access? What would help you understand this information better?	
7. Can you please describe the decision-making processes you use when choosing to take complementary medicine products?	

^a^Questions used to facilitate the IDIs and FGDs have been previously published in a related paper [33]; this current paper reports on another set of findings from the in-depth interviews and focus group discussions

In-depth interviews and focus group discussions were held over an eight-month period, from February to October 2016. Participants received an information sheet and had the opportunity to discuss the study and their possible participation with the lead author before consenting to participate. All participants signed consent forms before participating in an IDI or FGD. Consent was also confirmed orally and audio-recorded before FGDs and IDIs commenced. Participation was voluntary, and women could choose to withdraw from the study at any time. Participants could choose to participate in either an IDI or a FGD, and if an IDI, choose whether this occurred face-to-face, over the telephone or Skype, according to what was most convenient for them, and their family and work commitments. The lead author conducted all interviews and focus groups.

Demographic details and data on women’s use of CMPs at the time of the interview and in the previous 12 months were also collected.

Women’s health literacy levels were measured using two validated health literacy screening tools completed verbally and individually before participating in IDIs or FGDs. The first was the standard single question health literacy measure ‘*How confident are you filling out medical forms by yourself?’*, which uses a scale of responses to identify consumers with inadequate health literacy [41]. The second was the *Newest Vital Sign*, a three-minute direct test of participants’ abilities [42]. This identifies people with potential limitations in functional health literacy by measuring reading ability and interpretation skills, and aspects of numeracy necessary to understand nutritional information on food labels [42, 43].

### Data analysis

Descriptive statistics were used to analyse the results from the demographic survey and health literacy assessment tools. Responses to the single-item health literacy measure were recorded from the options ‘extremely’, ‘quite a bit’, ‘somewhat’, ‘a little bit’ and ‘not at all’. Those that chose ‘somewhat’, ‘a little bit’ or ‘not at all’ were considered to be at risk of limited health literacy [41, 44]. For the *Newest Vital Sign* assessment, participants who answered four or more of the six questions correctly were considered to have adequate functional health literacy; a score of less than four indicated possible limited functional health literacy; and a score less than two indicated that the participant had a large (> 50%) chance of having inadequate health literacy skills [42].

Transcriptions of all IDIs and FGDs were checked for accuracy by the lead author. Braun and Clark’s [45] method of thematic analysis was used to identify patterns and themes across the transcripts. The transcripts were read repeatedly and thoroughly, initial codes generated and organised into general themes which were then reviewed, defined and named in an inductive process [45, 46]. The NVivo10 program was used to code transcripts. LB1 coded all transcripts, and PA coded several. Both authors discussed identified themes and subthemes for the final analysis. Data from IDIs and FGDs were compared, as were data from pregnant versus breastfeeding women. The data from the whole sample were analysed together because there were no evident differences. Confidentiality was ensured by de-identifying transcripts and assigning pseudonyms to participants.

## Results

In total 25 women participated in the study; seven were currently pregnant, 17 were currently breastfeeding and one was both currently pregnant and breastfeeding. For the 18 breastfeeding participants, the age of their youngest breastfed child varied from 2 weeks to 21 months (mean age 7.76 months). Interviews took 40–60 min and focus groups lasted for 70–90 min with two to four women participating. Sixteen in-depth individual interviews (nine face-to-face and seven over Skype) and three face-to-face focus groups were held over a six-month period.

### Participant demographics

Participants ranged in age from 23 to 40 years, with 32.3 and 33 years being the average and median age, respectively. The sample contained both first-time and mothers with older children ranging in age from 2 to 11 years old. Mothers with older children all reported having breastfed these children for between 6 and 34 months. Eleven of the participants were health care practitioners, including three midwives, one nurse and seven allied health care practitioners. None of the participants currently smoked. Twenty-four women completed the two health literacy screening tests. Results for the standard item health literacy screening question showed that only two participants were at risk of limited health literacy. Results from the Newest Vital Sign demonstrated adequate functional health literacy for all but one participant. Other demographic data are presented in Table 2 [33].

**Table 2:** Demographic profile of the participants [33]^a^

Demographic Characteristics	Frequency (n)
Education	
Year 10 or equivalent	1
Year 12 or equivalent	2
Certificate 1–4 level^b^	4
Diploma^c^	2
Bachelor’s degree or postgraduate studies	16
Current employment status	
Full time home duties	3
On maternity leave from paid work	11
Part time employment	7
Full time employment	4
Income^d^	
Low household income (AUD $475–793 per week)	4
Medium household income (AUD $793–1814 per week)	9
Higher income (> AUD $1815 per week)	11
Prefer not to answer	1
Relationship status	
Married or de facto relationship	24
Single	1
Birthplace	
Australia	14
New Zealand or the United Kingdom	8
South Africa, the Netherlands, Colombia	1 each
Cultural and linguistic diversity	
Women who identified as being from non-English speaking backgrounds	4
Women who identified as being from English speaking backgrounds	21
Health literacy levels (*n* = 24 completed)	
Single item health literacy evaluation question: *how confident are you in filling out medical forms by yourself?*	
Extremely (adequate health literacy)	16
Quite a bit (adequate health literacy)	6
Somewhat (at risk of limited health literacy)	2
Newest Vital Sign	
Adequate functional health literacy (score ≥ 4 correct items out of 6)	23
Limited functional health literacy (score 3/6)	1

^a^Demographic data from this group of participants has been previously published [33]; this current paper reports on another set of findings from the in-depth interviews and focus group discussions

^b^Certificate 1–4 courses are provided through the Vocational Education and Training sector in Australia, and cover industry-specific knowledge and skills involving communication, teamwork, literacy and numeracy [47]

^c^Diplomas are provided through the Vocational Education and Training sector in Australia and provide education for working in industry, enterprise and paraprofessional careers [47]

^d^According to Australian Bureau of Statistics 2015–16 income distribution quintiles [48]

### CMPs used in pregnancy and lactation

All participants were currently taking CMPs when they participated in the study, and many reported previous use during pregnancy and/or breastfeeding (Additional file 2). Pregnancy and breastfeeding multivitamin preparations were the most frequently reported across participants followed by probiotics. All 25 participants reported either currently or previously taking pregnancy or breastfeeding multivitamins. Essential fatty acid supplements were also popular, with a total of 18 women reporting having taken these at some point during pregnancy. Ten breastfeeding women reported currently taking probiotics, and 18 women reported using probiotics either currently, or at some point during pregnancy. More breastfeeding women (10/25) reported currently using herbal medicines than pregnant women (5/25).

Participants’ overall reasons for taking CMPs were varied and have been discussed previously [33]. Desire for holistic health care, CMP use being culturally normal, and previous positive experiences with CAM or CMPs were frequently reported [33]. Regarding specific CMPs, women self-prescribed or took CMPs on the advice of their HCPs for various reasons. Vitamin and mineral supplements were taken to ensure adequate micronutrient intake to support their unborn and breastfeeding babies’ health and development, and/or their own health. Probiotics were used to optimise gut and digestive health in general, but also after antibiotic use (post-caesarean or after mastitis) to restore gut flora, and as a preventative or adjunct treatment to vaginal thrush (*Candida albicans)* during pregnancy or lactation, or nipple thrush and mastitis during breastfeeding. During breastfeeding, women frequently reported previous or current use of herbal medicines, either to treat or prevent common conditions of breastfeeding like blocked ducts or mastitis, or as galactagogues due to perceived or diagnosed breastmilk supply issues.

### Information sources on CMPs

The information sources participants used to find CMP information can be grouped into three main categories (Table 3).

**Table 3:** Information sources used by participants to find information on CMPs used in pregnancy and breastfeeding [33]^a^

Information source category	Specific information sources within the overall category	Number of participants who reported use of the specific information source^b^
(1) Information from health care practitioners (HCPs)	Trusted (primary) HCP	24
	Other HCPs: general advice	20
	CAM / Hospital Medication helplines	7
	Other HCPs: second opinion sought on specific CMPs	6
(2) Own and other pregnant or breastfeeding women’s experiences	Internet (google, blogs, social media)	22
	Own previous experience CMPs/CAM	21
	Other women’s experiences	19
	Intuition (used in final stages of the decision-making)	13
(3) Published research	Evidence-based quantitative research	13
	Hospital/University Databases	7
	Traditional Use	2
	Qualitative studies	1

^a^Information sources reported by this group of participants has been previously published [33]; this current paper reports on another set of findings from the in-depth interviews and focus group discussions

^b^Numbers are not mutually exclusive - participants reported use of multiple information sources

### Factors that impacted women’s decisions to take or not to take CMPs during pregnancy or breastfeeding

**Fig. 1:**
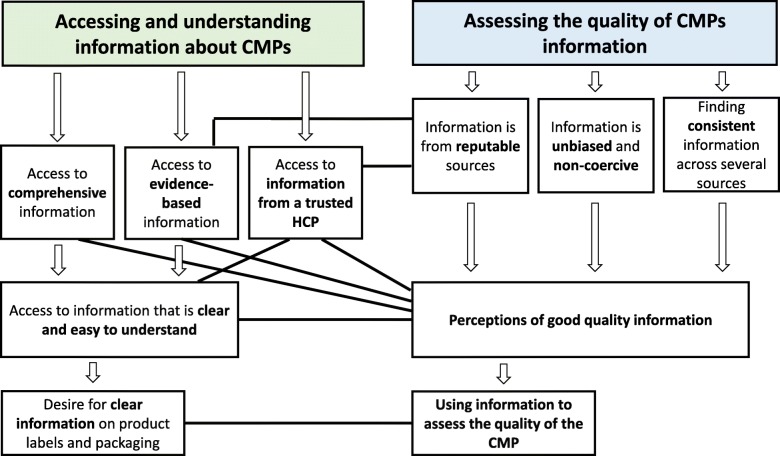
Coding tree illustrating the two major themes with interrelated subthemes

Two broad factors influenced the participants’ decisions to take, or not to take, a CMP. These can be conceptualised into two major themes with interrelated subthemes: (i) *Accessing and understanding information about CMPs;* and (ii) *Assessing the quality of CMPs information* (Fig. 1). Theme 1 was central to women’s search for whether a clear benefit to taking a CMP when pregnant or breastfeeding could be established. Theme 2 encompasses subthemes that describe how the participants assessed the quality of information and information sources during decision-making, and how they used these assessments to evaluate the quality of CMPs they considered using.

**Fig. 2:**
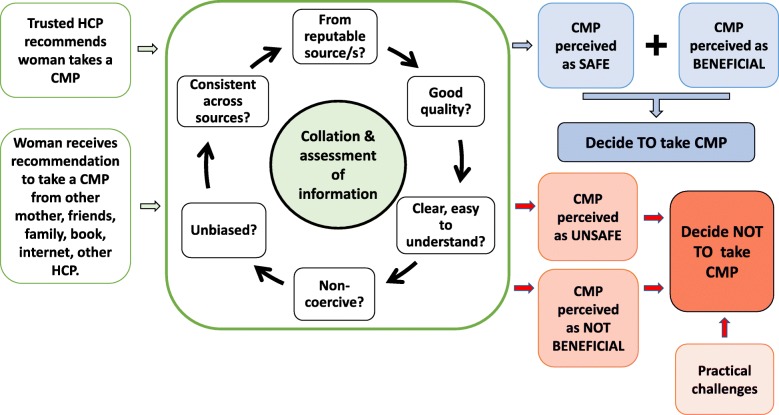
Participants’ reiterative collation and assessment of information during their decision-making processes

The participants were driven by the need to find understandable information on the CMPs they considered using, and engaged in a reiterative process of collating and assessing this information (Fig. 2). This process occurred whether the recommendation to take a CMP came from a HCP or lay source. Their overall aim was to identify whether safety and possible benefits to taking a CMP during pregnancy or breastfeeding could be established before making a decision. Participants also identified some practical challenges that led them to decide not to take a CMP. These included experiencing nausea or difficulty in swallowing tablets or capsules, disliking the taste of supplements or herbal medicines, the cost of CMPs and knowing they were likely to forget to take a CMP if they purchased it. Practical challenges however were minor considerations in women’s decision-making processes compared with the reiterative collection and assessment of information to establish the benefits and safety of CMPs.

### Accessing and understanding information about CMPs

The participants wanted access to easy to understand, comprehensive information, preferably given to them by their most trusted HCP. Quite a few also spoke about searching for evidence-based information. These elements contributed to the participants’ perceptions of good quality information (Fig. 1). All wanted clear information on product labels and packaging, and many wanted more comprehensive written information to be supplied with CMPs, or to be easy to access.

### Access to comprehensive information

Some participants did report receiving written information from their HCPs but most wanted more comprehensive information regarding benefits and safety considerations from their HCPs.

*There wasn’t anything from the hospital about complementary medicines. You imagine that that would be a good resource because it’s come from the midwife… to have some information provided on herbs contraindicated in pregnancy and breastfeeding, rather than having to go and seek it out myself. (Zilla, breastfeeding mother)*

The participants felt that having comprehensive information was critical in influencing their decisions, and this need drove their searches to establish safety or benefits of CMPs.

*I called up the company and they had a naturopath you could speak to. My first question was ‘Can I take this [CMP] while breastfeeding?’ Safety is the first thing I think about. I’ve been in pharmacies and I will ask them for information too, and call my naturopath, and also do more research. (Elise, breastfeeding mother)*

Women read product labels as well as written and on-line information and discussed CMPs information with HCPs and peers. If they felt information was incomplete, they would either seek more information before making a final decision or immediately decide not to take a CMP, preferring to err on the side of caution.

*I want to know what it’s for and what the side effects are. The peppermint water, I got it made up by the pharmacist so there was no written information. I spoke to the pharmacist and the lactation consultant about it, and she gave me an article on it, but then I also Googled it to get information before deciding to use it. I think written information is really important to go along with it. (Penelope, breastfeeding mother)*

### Access to evidence-based information

Scientific evidence was important to quite a few women, including the health care practitioners and some of the laywomen in the sample. The participants wanted to receive information on safety and benefits from reputable sources including published research and government-endorsed websites. Over half of the sample looked for evidence-based research, referring to PubMed, Cochrane, university and hospital databases as resources, as well as wider ‘Google’ searches (Table 3).

*I want to avoid a caesarean this time and have a midwifery-led birth. My midwife alerted me to a particular study, about using [CMP] to help progress labour …I had a look at the study to see what it was, where it was conducted, how many women, and what benefits there were. And so now I’ve been taking the [CMP] since 37 weeks [gestation]. (Donna, pregnant mother)*

Lack of scientific evidence caused uncertainty for some participants and led to decisions not to take a CMP.

*Complementary medicines often come up as unsafe to take when you’re pregnant or breastfeeding. And then when you actually look into it, often it’s just that there have been no studies done, it’s just unknown. But if it hasn’t been studied, then you think ‘Maybe I shouldn’t take it then because it could have some effect on my baby that I don't know about.’ (Kim, breastfeeding mother)*

### Access to information that is clear and easy to understand

Women’s decisions were influenced by their ability to understand the information they gathered. Even the health care practitioners in the cohort preferred information that was easy to understand, whether it was written on labels, pamphlets or received hand-written from a HCP. If information was insufficient or difficult to understand, the participants reported deciding not to take a CMP. Conversely, some women relied on information from labels on packaging to choose the CMPs marketed towards pregnant or breastfeeding women, or when making sure specific ingredients were included in the CMP.

*I’m taking a prenatal vitamin with DHA, the fish oil. It is better [than other prenatal vitamins] because it also has the DHA for the development of the baby. I read all the labels and chose this one, as it has all the ingredients that I need for pregnancy. (Gabriela, pregnant mother)*

### Assessing the quality of information

Assessing the quality of the information collected was an important part of the participants’ decision-making (Fig. 1) and was a reiterative process (Fig. 2). Quality of information was assessed in several ways: by how much women trusted the information source and felt it was reputable, by finding the same information consistently across several sources, and by assessing whether the information was non-biased and non-coercive. Information was also perceived as good quality if it was clear and easy to understand. If conflicting or inconsistent information was found, and/or the information assessed was perceived as poor quality, difficult to understand or coercive, the participants would either decide against taking a CMP, or seek further information regarding safety and possible benefits before making their final decisions. If information was perceived as good quality, clear and easy to understand, they were more likely to take a CMP, provided they could also identify a clear benefit to taking the CMP (Fig. 2). However, regardless of how much information was collated, women would only take a CMP if the information could be used to establish a CMP’s safety for their unborn or breastfeeding baby, and their own health.

### Using reputable information sources and consistency of information across several sources

Women felt that information was good quality if it came from HCPs they knew and trusted. Some women preferred the CMPs recommended by their HCPs, or CMPs that were only available through HCPs rather than supermarket brands.

*If it is something more important like pregnancy, I trust my naturopath… I get her advice and buy them through her, because I know that she's chosen the best brands for her patients. (Elise, breastfeeding mother)*

Conversely, if they did not get information from trusted HCPs, it was important to them to find consistent information on a CMP from several other sources.

*I like to feel like I am getting the same information from lots of different sources. If you’re only getting information from one source, you don’t trust it as much, and you start to go ‘ok, well, this isn’t maybe what I need.’ (Willow, pregnant mother)*

Consistency of information was especially important when looking at information across several websites, but also when assessing information from known and unfamiliar HPCs as well as other mothers, friends, family, and books.

*The main thing is making sure that it’s recommended as being safe for the baby and then, weighing up the positives and if there are any negatives. And just talking to people, to a mother who’s been through it before, a naturopath or a pharmacist, a herbalist, wherever you’re buying your medicine from. (Joni, breastfeeding mother)*

Women assessed the trustworthiness of Internet information in several ways: Finding consistent information across several websites, assessing the authors’ professional qualifications, and sorting personal opinion sites from those endorsed by the Australian government, associated with professional bodies, or linked to research done through universities or hospitals.

*A good website is anything that’s government run... mothers’ groups, blogs, chat rooms, they’re fine to get ideas but I wouldn’t take that as reputable information. Research papers - so I will look up like Cochrane Reviews and things like that. (Penelope, breastfeeding mother)*

Women were aware of inconsistencies and inaccuracies in advice received from peers, the Internet and even health care practitioners. They felt it was their job to sort through it to find the truth:

*Receiving advice is like receiving a bag of second-hand clothes, knowing that there’s a Gucci handbag in the bottom. You’ve just got to sort through all the crap to get to that Gucci handbag. (Olivia, pregnant mother)*

### Assessing whether information was coercive or biased

Women wanted information about CMPs free from bias and advertising and would seek further information, dismiss or not take a product if the information was perceived as overtly marketing-based.

*I want clearer information, less marketing. In magazines, in the shops, it’s actually about selling the products, advertising, rather than how effective the product is. (Donna, pregnant mother)*

*I look at the information and ask, ‘Why are they promoting that? Is it because of personal experience, or is it because they're wanting to make money out of it, or is it because it's worked for them, but they haven't actually tried on it on anyone else?’ (Marley, breastfeeding mother)*

### Using the information to assess the quality of the CMP itself

Participants also discussed comparing strengths of different supplements when deciding between brands, recognising that cheaper options may not have equivalent strengths or quantities of active ingredients.

*I read the label [and] the strengths as well, and how many tablets you’ve got to take, because you might see ‘this is a hundred tablets, and it's really cheap’, but then you might have to take four tablets a day instead of two!’ (Elise, breastfeeding mother)*

Personal positive experience with particular brands also influenced the choice to use particular CMPs. Ethical issues including how ingredients were sourced and manufactured were also important to several participants and contributed to their evaluation of the quality of a CMP.

*I assess quality from previous knowledge, talking to a naturopath and doing my own little bit of research on the quality of the brands. …And just reading up things on the Internet about who the companies are, how they do their work, and studies. I want to know whether they are pharmaceutically tested, what high quality ingredients they're actually using, where they're sourcing them from. (Donna, pregnant mother)*

## Discussion

This study aimed to identify factors that impact pregnant or breastfeeding women’s decisions to take or not to take CMPs. The factors centre on how well women could access and understand information on CMPs, and how they assessed the quality of the information they gathered. The participants’ high health literacy levels are a limitation in this study, and may reflect the self-selected sampling and help explain their complex information-seeking and decision-making processes. However, it is useful to discuss health literacy and decision-making further. Fundamental to the factors involved in these women’s decisions to take, or not to take, a CMP were their health literacy levels, their use of multiple information sources, and their assessment of information gathered during their decision-making.

### Health literacy and information-seeking behaviour

The ability to seek out, appraise and understand health information is essential to good health literacy [27]. People with higher levels of education, income, and health literacy are more likely to search for health information [9] than other members of the population. Previous research has found higher education and current employment to be significantly correlated with CMP use in pregnancy by Australian women [1]. It is not surprising then, that the participants in this study were active health information seekers, as their demographic profile matches both what is known about Australian women who use CMPs in pregnancy [11, 31, 49], and what is known about people who actively engage in health information-seeking behaviours.

Another component of good health literacy demonstrated by participants in this study, was communicative health literacy, the ability to discuss collated health information with HCPs in order to apply relevant information to decision-making regarding their own, and their unborn or breastfeeding babies’ health [33, 50]. Constructive discussions of health information between patients and HCPs is considered a core component of shared decision-making [9]. In turn, shared decision-making can increase patient satisfaction if consumers believe they are fully informed and involved in their treatments [51], and improves the quality of the decision-making process and the consistency between patients’ values and choices [52]. However, as noted earlier, most of the participants wanted more comprehensive CMPs information than their HCPs provided, and so searched further to answer questions regarding the safety profiles and possible benefits of CMPs. Previous research has noted that many biomedical HCPs may not feel adequately informed to discuss complementary medicine with their patients [53], simply do not ask about CMP use [49, 54, 55], or discussion does not occur because women do not disclose CMP use, because they do not think it necessary [56] or because they worry that these HCPs will react negatively [14, 32, 53, 54]. It may be that some of the women’s biomedical HCPs were not adequately informed about CMPs, and so were unable to provide the amount of comprehensive information that the participants wanted. Conversely, the health information-seeking behaviours of this unique sample may simply illustrate their high health literacy skills.

The transition to motherhood begins at an early stage in pregnancy, with women being acutely aware of possible threats to their pregnancies and continues through the postpartum period [52] where women will make health care decisions that incorporate considerations of their breastfed infants’ safety [17]. The active information-seeking by participants in the present study reflects both their acute concern for the health of their unborn and breastfeeding babies, and their high health literacy levels. Information-seeking behaviours are associated with various outcomes including consumers wishing to increase their knowledge of treatment options and to discuss the results of their searches with their health care providers [9, 57], self-diagnosis, high self-efficacy with regards to self-management of health [58], and intentionally choosing between different health options [59]. Again, the reiterative collation and assessment of CMPs information shown in this study (Fig. 2) is an example of this, as women actively evaluated possible benefits and harms of CMPs in order to play an active role in their health care and make informed health care decisions.

### Use of multiple information sources

Higher health literacy is also associated with seeking and using a number of information sources rather than relying solely on health care practitioners to provide information on health and health care choices [57], as was demonstrated in this study (Table 3). Use of multiple information sources to gather information on medications has been noted internationally [60] and in general Australian consumers [57]. Studies of maternal health care show that women most commonly access health care practitioners for information on pharmaceutical and complementary medicines, and general health information [57, 60–62]. Lay sources (e.g. family members, friends, or pregnant or breastfeeding peers) are accessed for information on remedies to treat everyday health challenges and for emotional support [57], possibly due to shared cultural knowledge and norms [61]. The Internet is a frequently accessed resource [60, 61], but is mainly used to supplement, rather than provide alternate advice to that given by women’s health care practitioners [57]. Concerns have been raised regarding the reliability, accuracy and currency of information pregnant women may find on the Internet and use to support their health [63, 64]. Women in the present study recognised the need to be discerning when assessing information accessed via the Internet or social media, choosing to combine information found online with information from other mothers, HCPs and their own knowledge. Very little research has been published regarding mothers’ use of the Internet to find breastfeeding information and support [65, 66]. Other research however has also shown that women are cautious regarding the reliability of pregnancy information found on the Internet, preferring consistency of information across multiple sites [63, 67], or information that was linked to institutional websites and referenced scientific studies [63, 67].

### Understanding CMPs information

It is well-known that the receipt of poor quality or easily misunderstood health information can negatively influence consumers’ health care decisions [27, 68]. The participants’ preference for plain language on labels, written instructions and in verbal information received when purchasing CMPs confirms plain language is more easily understood than technical or medical information that uses jargon, acronyms or unnecessary words [27, 51]. Plain language is recommended in Australian [27] and international [51, 69] guidelines to improve consumers’ health literacy. Product labels and written information provided with a product need to be relevant to consumers, readable and appropriate if consumers are going to be able to use CMPs safely and effectively [70]. Women in this study wanted comprehensive written information to be supplied with their CMPs.

Also evident from this small study, was that participants were not always satisfied with the amount of information they found on labels and packaging and often searched more widely before making final decisions about a CMP. Women did appreciate receiving written CMPs information from their health care practitioners. Health care practitioners could possibly mitigate women’s needs to search widely for CMPs information by becoming familiar with, and knowledgeable about CMPs commonly used in pregnancy and lactation, including where to find evidence-based information. Providing comprehensive written information in plain language when recommending or providing CMPs to their patients [71] and initiating open, non-judgemental discussions with women regarding CMP use in pregnancy and lactation would also help facilitate women’s decision-making. Regarding women’s frequent use of the Internet to find health information, it may also be prudent for health care practitioners to guide women toward online resources linked to government, hospital or other respected health organisations [67]. This would both acknowledge and support women’s right to autonomy and involvement in health care choices while also helping them find reputable Internet resources.

### Assessing the quality of information during the decision-making process

The reiterative process of collating and assessing information noted in this study described not only the types of CMPs information pregnant or breastfeeding women wanted, but also how they assessed the quality of information received. It is difficult to compare this result with other research on CMP use in pregnancy and lactation as the ways mothers evaluate CMPs information is not well established. Most studies focus on the range of professional and lay information sources women access and encourage biomedical health care practitioners to ensure their patients receive scientifically validated information on CMPs (e.g. see [31, 72]). Future research could further investigate how women specifically evaluate and assess the quality of CMPs information received from health care practitioners, lay and Internet sources.

### Strengths and limitations

The participants were comparatively homogenous concerning education, income, English proficiency and health literacy levels. However, this can be considered both a strength and a limitation. The participants were highly health literate and so the data gathered is not representative of the full spectrum of health literacy levels. Additionally, nearly half the participants were health care professionals, which also influenced the health literacy of the sample. Although qualitative research is not intended to be generalised outside the study sample population, further research with women with lower health literacy levels is needed. However, the high health literacy of the participants is also a strength of this study, when considering how well the demographic profile of the participants matches what has been previously shown about typical Australian women who use CMPs in pregnancy [11, 31]. There may be problems finding Australian mothers with lower health literacy levels who use CMPs in pregnancy and lactation. Using a highly educated, health literate sample has generated in-depth insights into the information-seeking of these women who use CMPs in pregnancy and lactation.

### Implications for practice and future research

Health care practitioners need to be aware of the breadth of information sources pregnant and breastfeeding women will utilise when deciding whether to use a CMP. To have positive interactions with pregnant and breastfeeding women who use CMPs, HCPs would also benefit from learning about commonly used CMPs, including being able to adequately address women’s identified information needs concerning whether possible benefits or safety concerns can be identified. Being able to provide clear and comprehensive information, and to direct women to reputable online resources, as discussed above, would also be of benefit to HCPs’ professional practice skills, and the women for whom they care.

Future research needs to investigate whether women with lower health literacy levels use CMPs in pregnancy and lactation, and if so, whether similar factors influence their decision-making. Additionally, while this study did include four women who identified as being from non-English speaking backgrounds (Table 2), further research with Australian women from more varied language and cultural backgrounds is also warranted, and would be more reflective of the diverse Australian population.

## Conclusions

This study showed that participants’ high health literacy levels led them to engage in reiterative, information-seeking processes fuelled by the need to find clear information before making the decision to take, or not to take, a CMP. Two main factors influenced women’s decisions: 1) how well they understood information gathered on CMPs; and 2) how they assessed the quality of the information collated, including how they used this to assess the quality of a CMP itself. Decisions *to take* a CMP depended on women perceiving a clear benefit to their baby or themselves, establishing safety of the CMP in pregnancy and breastfeeding, and perceptions of the CMPs information collated as good quality and from reputable sources. Conversely, final decisions *not to take* a CMP resulted when women could not identify an obvious benefit to taking a CMP, were unable to establish the safety of a CMP, and/or when they perceived CMPs information as poor quality, difficult to understand or coercive.

## Additional files

Additional file 1: COREQ: Consolidated criteria for reporting qualitative studies (COREQ): 32-item checklist. *Submitted for the paper by Barnes, Barclay, McCaffery and Aslani (2019) ‘Factors influencing women’s decision-making regarding complementary medicine product use in pregnancy and lactation’.* COREQ Checklist in table form for the study. (PDF 697 kb)

Additional file 2: Types of CMPs used in pregnancy and lactation. Figure in cluster column graph form, illustrating complementary medicine product use during pregnancy and breastfeeding as reported by the participants. (PDF 593 kb)

## Data Availability

The datasets generated and analysed during the current study are not publicly available as participants did not consent to transcripts of interviews and focus group discussions being shared. Additional details relating to other aspects of the data are available on reasonable request from the authors.

## Abbreviations

Biomedical HCPs: Biomedical health care practitioners. (Biomedically trained health care practitioners - nurses, midwives, doctors and obstetricians)
CAM HCPs: Complementary medicine health care practitioners. (Western complementary medicine health care practitioners including naturopaths and herbalists trained in Western Herbal Medicine, and Traditional Oriental Medicine practitioners)
CAM: Complementary and alternative medicine
CMP: Complementary medicine product
CMPs: Complementary medicine products
FGDs: Focus group discussions
HCP: Health care practitioner
HCPs: Health care practitioners
IDIs: In-depth interviews
