# Supplementary material for: Factors influencing women’s decision-making regarding complementary medicine product use in pregnancy and lactation
Source: BMC Pregnancy Childbirth. 2019 Aug 7;19:280. doi: 10.1186/s12884-019-2396-2 (PMC6686446; doi:10.1186/s12884-019-2396-2)
Supplement: Supplementary file 1 — COREQ: Consolidated criteria for reporting qualitative studies (COREQ): 32-item checklist. Submitted for the paper by Barnes, Barclay, McCaffery and Aslani (2019) ‘Factors influencing women’s decision-making regarding complementary medicine product use in pregnancy and lactation’. COREQ Checklist in table form for the study. (PDF 697 kb) [file 12884_2019_2396_MOESM1_ESM.pdf]

COREQ: Consolidated criteria for reporting qualitative studies (COREQ): 32-item checklist

Submitted for the paper by Barnes, Barclay, McCaffery and Aslani (2019) 'Factors influencing women's decision-making regarding complementary medicine product use in pregnancy and lactation'

**Domain 1: Research team and reflexivity**	
Personal Characteristics	
1. Interviewer/facilitator<br><br>*Which author/s conducted the interview or focus group?*	The first author, Larisa Barnes, conducted all interviews and focus groups.
2. Credentials<br><br>*What were the researcher's credentials? E.g. PhD, MD*	Ms Larisa Barnes, BA, BNat(Hons), is a PhD candidate with The University of Sydney School of Pharmacy, Faculty of Medicine and Health.<br><br>Prof Parisa Aslani, BPharm(Hons), MSc, PhD, Grad Cert Ed Stud (Higher Ed), is the Professor in Medicines Use Optimisation, The University of Sydney School of Pharmacy, Faculty of Medicine and Health.<br><br>Prof Lesley Barclay, A.O., PhD, is an Emeritus Professor of the University of Sydney, with the University Centre for Rural Health and The University of Sydney School of Public Health.<br><br>Prof Kirsten McCaffery, BSc (Hons), PhD, is a Professorial Research Fellow at The University of Sydney School of Public Health, Faculty of Medicine and Health.
3. Occupation<br><br>*What was their occupation at the time of the study?*	Larisa Barnes is a full-time PhD student and who also worked as a clinical educator in the Bachelor of Naturopathy program, supervising students on clinical placement at the Southern Cross University Health Clinic.<br><br>Professors Aslani and McCaffery are both employed as academics at The University of Sydney in the School of Pharmacy and School of Public Health, respectively. Professor Aslani is also a registered pharmacist. Professor Barclay worked as an Emeritus Professor for The University of Sydney and was the former Director (now retired) of the University Centre for Rural Health.
4. Gender<br><br>*Was the researcher male or female?*	All researchers are female. All are mothers.
5. Experience and training<br><br>*What experience or training did the researcher have?*	Larisa Barnes worked as a research assistant conducting focus groups and in-depth interviews and assisted with analysis of qualitative data for research projects at the University Centre for Rural Health and Southern Cross University since completing her

	Honours in 2004. Larisa has experience using phenomenological theory, symbolic interactionism and thematic analysis to analyse qualitative data.<br><br>Prior to this, Larisa Barnes completed a mixed-methods Honours degree that involved in-depth interviews and survey research. Larisa also worked as a practising naturopath from 2001-2016. This provided some contextual background to CMP use in pregnancy and lactation.<br><br>Professors Aslani, Barclay and McCaffery have extensive research experience spanning the fields of pharmacy, medicines use optimisation, public health, health literacy and maternity care and services provision. Professor Aslani also teaches in the School of Pharmacy at The University of Sydney and formerly worked as a pharmacist. Prof Barclay worked as a clinical midwife in the past and currently supports midwifery students doing higher degrees by research.
Relationship with participants	
6. Relationship established<br><br>*Was a relationship established prior to study commencement?*	*Face-to-face interviews and focus group discussions*<br><br>By attending some supported playgroups, pregnancy and breastfeeding support groups, and mothers groups, Larisa Barnes made contact with potential participants and invited participation. However, no formal relationship was established prior to the study with the participants. None of the participants were personal patients of Larisa Barnes or her students at the Southern Cross University Health Clinic where she worked as a clinical educator during the time of data collection.<br><br>*Skype and telephone interviews*<br><br>Women who participated in Skype and telephone interviews generally lived at a distance to Larisa Barnes, in metropolitan Sydney, or South-East Queensland. They learned about the study through snow-ball recruitment and electronic advertising. No formal relationship was established with these participants prior to the study.
7. Participant knowledge of the interviewer<br><br>*What did the participants know about the researcher? e.g. personal goals, reasons for doing the research*	Larisa Barnes made it clear to the participants that in addition to being a PhD student, she was a naturopath and a mother, and had birthed and breastfed two children. Larisa approached the topic of CMP use in pregnancy and lactation with respect for both complementary and biomedical health care systems and encouraged women to feel comfortable about speaking about their use of both or either. She explained to all participants that her reasons for doing the research stemmed from her experiences during pregnancy, childbirth and breastfeeding, as well as her clinical experience as a naturopath, and love of

	research. Her goals were to try and understand other women's choices to use CMPs in pregnancy and breastfeeding, and how they found out their information about CMPs, and to share this information with the wider community through publications of her research. Further information about the study was also provided to the participants via the Participant Information Statement.
8. Interviewer characteristics<br><br>*What characteristics were reported about the interviewer/facilitator? e.g. Bias, assumptions, reasons and interests in the research topic*	No characteristics are reported about the interviewer/facilitator Larisa Barnes in the text of the paper, except her qualifications and institutional affiliations. However participants were made aware of Larisa's personal goals and reasons for doing the research (see 7. above).
**Domain 2: study design**	
Theoretical framework	
9. Methodological orientation and Theory<br><br>*What methodological orientation was stated to underpin the study? e.g. grounded theory, discourse analysis, ethnography, phenomenology, content analysis*	No specific theories underpinned the study design or interview questions. After data collection was completed, thematic analysis was used to analyse the data, following an interpretive qualitative approach. Transcripts were analysed using thematic analysis as described by Braun and Clarke[1] using several steps.<br>i. Becoming familiar with the data by repetitively reading it throughout the coding process<br>ii. Identifying initial concepts or codes and pooling data relevant to each code<br>iii. Collating codes into potential themes<br>iv. Gathering all data relevant to each theme<br>v. Reviewing themes<br>vi. Generating clear definitions and names for each theme<br><br>The manuscript summarises this process by stating:<br><br>All transcripts were read on many occasions to ensure a thorough understanding of the themes as they emerged and were grouped into major and minor subthemes. As no notable differences appeared between data from the focus group discussions and in-depth interviews, and between pregnant versus breastfeeding women, the data from all participants was grouped together for analysis.
Participant selection	
10. Sampling<br><br>*How were participants selected? e.g. purposive,*	Purposive sampling followed by snowball sampling approaches were used for recruitment and was directed at pregnant and breastfeeding women who used CMPs.

convenience, consecutive, snowball	
11. Method of approach<br><br>*How were participants approached?* e.*g. face-to-face, telephone, mail, email*	The study was advertised on posters and flyers at playgroups, antenatal classes, pregnancy and postnatal yoga classes, pregnancy and parenting support groups, and in pharmacies and allied health practices; and on free local classified advertising networks and through Sydney University electronic media channels. Participants then approached Larisa Barnes via email or telephone if they were interested in participating.<br><br>For the Northern Rivers area of NSW, Larisa Barnes visited various playgroups, antenatal classes, pregnancy and postnatal yoga classes, pregnancy and parenting support groups to inform potential participants about the study and to leave flyers and posters. Again, participants then approached Larisa Barnes via email or telephone if they were interested in participating. A few also approached her in person on the days she visited regarding participation after they read the information flyers.
12. Sample size<br><br>*How many participants were in the study?*	Twenty-five women (n=7 pregnant, n=17 breastfeeding, n=1 both pregnant and breastfeeding) participated in the study.
13. Non-participation<br><br>*How many people refused to participate or dropped out? Reasons?*	Although originally 29 women agreed to participate, four women withdrew from the study. One woman went into labour the day before her interview. Another woman asked to delay her interview until her newborn was a bit older and she felt more comfortable going out and about with an infant. Two women did not give reasons for not attending their interview or focus group sessions.
Setting	
14. Setting of data collection<br><br>Where was the data collected? e.*g. home, clinic, workplace*	*Face-to-face in-depth interviews and focus group discussions*<br><br>Face-to-face interviews and focus group discussions took place at public venues familiar to the participants, that were baby and toddler-friendly so participants could comfortably breastfeed and look after their children. These were meeting rooms in public libraries and community centres and playgroup venues. Interviews and focus groups were held in these venues at separate times to normal activities like playgroups and story-time, so the venues were quiet.<br><br>*Skype and telephone interviews*<br><br>Women who participated in Skype and telephone interviews usually participated from their homes. One participant rang from her private office on her lunchbreak at work.

15. Presence of non-participants<br><br>*Was anyone else present besides the participants and researchers?*	Women were welcome to bring their babies and toddlers to the interviews or focus group discussions if they wished, especially as finding childcare could be a barrier to participation, and in recognition that breastmilk would probably be the sole source of nutrition for breastfed babies 6 months and under, and the main source of nutrition for breastfed babies up to 12 months. Some women chose to bring their babies and toddlers with them, and others chose to attend without their children.
16. Description of sample<br><br>*What are the important characteristics of the sample? e.g. demographic data, date*	All women reported currently taking CMPs, or having previously taken them in the last month. Participants ranged in age from 23 years to 40 years, the average age was 32 years. None of the women identified as smokers. Around half the sample were first-time mothers. Fourteen mothers had between one and four older children, ranging in age from two to eleven years old. All women with older children reported having breastfed their older children for between 6 and 34 months, with the average time being 18 months. Other demographic data is summarised in Table 2.<br><br>Data was collected over a 6 month period between March and October 2016.
Data collection	
17. Interview guide<br><br>*Were questions, prompts, guides provided by the authors? Was it pilot tested?*	A seven-item semi-structured interview guide was used during focus groups and interviews. This can be seen in Table 1. The interview guide was pilot-tested. Feedback from pre-testing for face and content validity from an interview with one pregnant woman, and a focus group with one pregnant and three breastfeeding women, was used to refine the questions.
18. Repeat interviews<br><br>*Were repeat interviews carried out? If yes, how many?*	No repeat interviews were carried out.
19. Audio/visual recording<br><br>*Did the research use audio or visual recording to collect the data?*	All in-depth interviews and focus group discussions were audio-recorded and transcribed by an independent transcription service, then checked for accuracy against the original recording by the lead author.
20. Field notes<br><br>*Were field notes made during and/or after the interview or focus group?*	The lead author facilitated all interviews and focus groups and also kept a detailed research journal where ideas and themes from each interview and focus group were documented in an ongoing iterative process.
21. Duration<br><br>*What was the duration of the interviews or focus group?*	Interviews lasted for 40-60 minutes; focus groups 70-90 minutes.

22. Data saturation<br><br>*Was data saturation discussed?*	Final sample size was determined by thematic saturation, which was reached at 22 participants. An additional three interviews were held to confirm that no new themes were apparent in this new data.
23. Transcripts returned<br><br>*Were transcripts returned to participants for comment and/or correction?*	Transcripts were not returned to participants for comment or correction.
**Domain 3: analysis and findings**	
Data analysis	
24. Number of data coders<br><br>*How many data coders coded the data?*	Two people coded the data – Larisa Barnes (LAJB) and Parisa Aslani (PA).<br><br>Transcripts were analysed using thematic analysis as described by Braun and Clarke[1] and using the NVivo10 program by the lead author (LAJB). All transcripts were read on many occasions to ensure a thorough understanding of the themes as they emerged and were grouped into major and minor subthemes. To increase validity PA coded several transcripts and both authors discussed and agreed on identified themes and subthemes for the final analysis.
25. Description of the coding tree<br><br>*Did authors provide a description of the coding tree?*	The coding tree is described in the following paragraph (from Thematic Analysis in Results section) and illustrated in Figure 1.<br><br>*Two broad factors influenced the participants' decisions to take, or not to take, a CMP. These can be conceptualised into two major themes with interrelated subthemes: (i) Accessing and understanding information about CMPs; and (ii) Assessing the quality of CMPs information (Figure 1). Theme 1 was central to women's search for whether a clear benefit to taking a CMP when pregnant or breastfeeding could be established. Theme 2 encompasses subthemes that describe how the participants assessed the quality of information and information sources during decision-making, and how they used these assessments to evaluate the quality of CMPs they considered using.*
26. Derivation of themes<br><br>*Were themes identified in advance or derived from the data?*	Themes were derived from the data.
27. Software<br><br>*What software, if applicable, was used to manage the data?*	Transcripts were analysed using thematic analysis as described by Braun and Clarke[1] and using the NVivo10 program by the lead author (LAJB).

28. Participant checking<br><br>*Did participants provide feedback on the findings?*	No.
Reporting	
29. Quotations presented<br><br>*Were participant quotations presented to illustrate the themes / findings? Was each quotation identified? e.g. participant number*	Selected quotations are presented to illustrate the themes and findings of the research. Quotations are identified with participant pseudonyms and their pregnancy or breastfeeding status, e.g. 'Donna, pregnant mother' or 'Zilla, breastfeeding mother'.
30. Data and findings consistent<br><br>*Was there consistency between the data presented and the findings?*	The quotations from the data were carefully chosen to illustrate the themes and findings of the research. We believe that there is consistency between the data presented and the findings of the research.<br><br>Please note, the findings were very rich and detailed. This paper focusses on the factors that influence participants' decision-making to take or not to take a CMP. Broadly, these factors encompass how they 1) accessed and understood CMPs information about CMPs, and 2) how they assessed the quality of CMPs information.<br><br>Two other papers reporting on the data will also be submitted for peer-review in relevant journals. These focus on the decision-making components to take CMPs in pregnancy or lactation as described by the participants, and on the information-seeking behaviours of the participants.
31. Clarity of major themes<br><br>*Were major themes clearly presented in the findings?*	Major themes are clearly presented in the text and in Figures 1 and 2. Relevant quotes from the participants also clearly illustrate the themes and interrelated sub-themes.
32. Clarity of minor themes<br><br>*Is there a description of diverse cases or discussion of minor themes?*	Subthemes for each major theme are discussed in the text and illustrated in Figure 1. The two major themes with their subthemes as they appear in the text of the Results section are below. Please note that the subthemes for each major theme are connected and interrelated, as illustrated in Figure 1, and incorporate the subthemes not included in the subtitles of the Results section, as indicated in italics below.<br><br>Accessing and understanding information about CMPs<br>• Access to comprehensive information<br>• Access to evidence-based information<br>• Access to information that is clear and easy to understand<br>• Text also illustrates subtheme *Access to information from a trusted HCP* and *Desire for clear information on product labels and packaging.*

	Assessing the quality of information<br>• Using reputable information sources and consistency of information across several sources<br>• Assessing whether information was coercive or biased<br>• Using the information to assess the quality of the CMP itself<br>• Text also illustrates subtheme *Access to information from a trusted HCP* and *Perceptions of good quality information*

Reference cited:

1. Braun V, Clarke V. Using thematic analysis in psychology. Qualitative Research in Psychology. 2006;3(2):77-101.
