# Supplementary material for: Factors influencing women’s decision-making regarding complementary medicine product use in pregnancy and lactation
Source: BMC Pregnancy Childbirth. 2019 Aug 7;19:280. doi: 10.1186/s12884-019-2396-2 (PMC6686446; doi:10.1186/s12884-019-2396-2)
Supplement: Supplementary file 2 — Types of CMPs used in pregnancy and lactation. Figure in cluster column graph form, illustrating complementary medicine product use during pregnancy and breastfeeding as reported by the participants. (PDF 593 kb) [file 12884_2019_2396_MOESM2_ESM.pdf]

Additional file 2. Types of CMPs used in pregnancy and lactation

As reported by participants in the study by Barnes, Barclay, McCaffery and Aslani (2019) Factors influencing women's decision-making regarding complementary medicine product use in pregnancy and lactation, BMC Pregnancy and Childbirth.

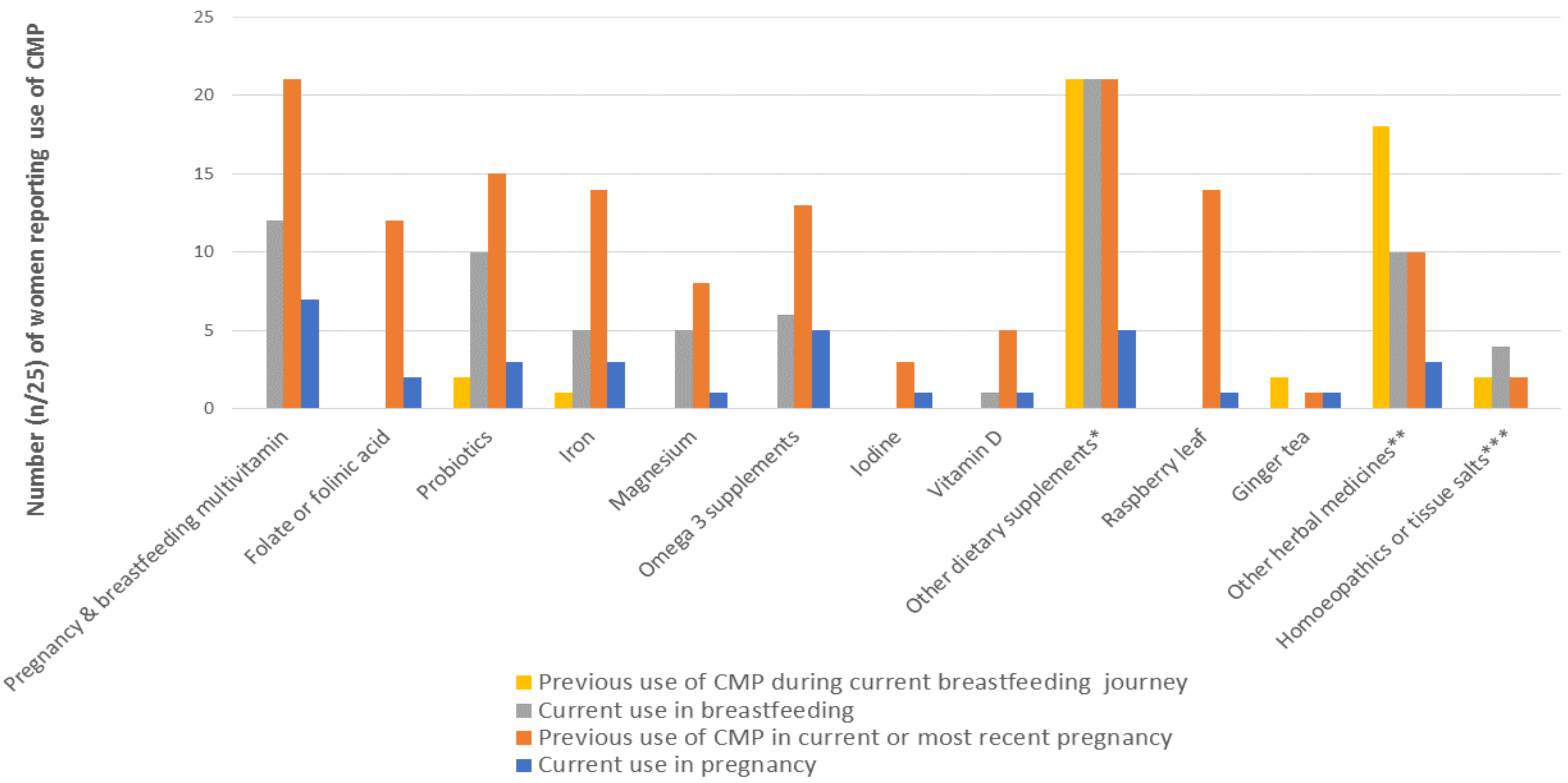

**Other dietary supplements included Evening primrose oil, Calcium, Magnesium, Vitamins C, B complex and B6, Zinc, Selenium, Lecithin, Brewer's yeast, a mixed vitamin-mineral thyroid support tablet, Glutathione, Calcium di-gluconate*

*** Other herbal medicines included herbal extract blends and supplements individually prescribed by participants' HCPs for various health conditions, several different herbal teas taken to support breastfeeding (supply and to treat / prevent mastitis), and/or to support digestion*

**** Used to treat nausea, mastitis, for labour induction or unspecified reason*
